# MAF-net: multi-receptive attention fusion network with dual-path squeeze-and-excitation enhancement module for uterine fibroid segmentation

**DOI:** 10.3389/fphys.2025.1659098

**Published:** 2025-09-17

**Authors:** Yun Jiang, Qiquan Zeng, Hongmei Zhou, Xiaokang Ding

**Affiliations:** ^1^ Department of Obstetrics and Gynecology, Quzhou Hospital of Traditional Chinese Medicine, Quzhou TCM Hospital at the Junction of Four Provinces Affiliated to Zhejiang Chinese Medical University, Quzhou, China; ^2^ College of Mechanical Engineering, Quzhou University, Quzhou, China; ^3^ Department of Color Ultrasonic, Quzhou Hospital of Traditional Chinese Medicine, Quzhou TCM Hospital at the Junction of Four Provinces Affiliated to Zhejiang Chinese Medical University, Quzhou, China

**Keywords:** image segmentation, uterine fibroid, multi-receptive attention fusion module, dualpath squeeze-and-excitation enhancement module, dual-scale attention enhancement module

## Abstract

**Introduction:**

Uterine fibroids are one of the most common benign tumors affecting the female reproductive system. In clinical practice, ultrasound imaging is widely used in the detection and monitoring of fibroids due to its accessibility and non-invasiveness. However, ultrasound images are often affected by inherent limitations, such as speckle noise, low contrast and image artifacts, which pose a substantial challenge to the precise segmentation of uterine fibroid lesions. To solve these problems, we propose a new multi-receptive attention fusion network with dual-path SE-enhancement module for uterine fibroid segmentation.

**Methods:**

Specifically, our proposed network architecture is built upon a classic encoder-decoder framework. To enrich the contextual understanding within the encoder, we incorporate the multi-receptive attention fusion module (MAFM) at the third and fourth layers. In the decoding phase, we introduce the dual-scale attention enhancement module (DAEM), which operates on image representations at two different resolutions. Additionally, we enhance the traditional skip connection mechanism by embedding a dual-path squeeze-and-excitation enhancement module (DSEEM).

**Results and discussion:**

To thoroughly assess the performance and generalization capability of MAF-Net, we conducted an extensive series of experiments on the clinical dataset of uterine fibroids from Quzhou Hospital of Traditional Chinese Medicine. Across all evaluation metrics, MAF-Net demonstrated superior performance compared to existing state-of-the-art segmentation techniques. Notably, it achieved Dice of 0.9126, Mcc of 0.9089, Jaccard of 0.8394, Accuracy of 0.9924 and Recall of 0.9016. Meanwhile, we also conducted experiments on the publicly available ISIC-2018 skin lesion segmentation dataset. Despite the domain difference, MAF-Net maintained strong performance, achieving Dice of 0.8624, Mcc of 0.8156, Jaccard of 0.7652, Accuracy of 0.9251 and Recall of 0.8304. Finally, we performed a comprehensive ablation study to quantify the individual contributions of each proposed module within the network. The results confirmed the effectiveness of the multi-receptive attention fusion module, the dual-path squeeze-and-excitation enhancement module, and the dual-scale attention enhancement module.

## 1 Introduction

Uterine fibroids are a common type of benign tumor that occurs within the uterus of women. Their incidence rate among women of childbearing age is as high as 70%–80% (Wang et al., 2024). Traditionally, hysterectomy has always been the most commonly used treatment method. Although this method can completely eliminate uterine fibroids and prevent their recurrence, its operation process is highly invasive and often leads to irreversible physiological consequences. In recent years, advancements in non-invasive therapeutic technologies have led to the emergence of high-intensity focused ultrasound (HIFU) as a promising alternative. HIFU offers several clinical advantages, including targeted ablation of fibroids without incisions, reduced postoperative complications, shorter recovery times, and preservation of uterine function. Whether it is the traditional surgical therapy or the high-intensity focused ultrasound therapy, an accurate preoperative assessment of the characteristics (size, number and anatomical location) of uterine fibroids remains crucial. Among available diagnostic tools, ultrasound imaging stands out as the most accessible, cost-effective, and widely used technique for the detection and localization of uterine fibroids. However, achieving reliable and precise segmentation of fibroids from ultrasound images remains a significant challenge in clinical practice. Currently, segmentation tasks are predominantly performed manually by experienced radiologists or sonographers. This manual process is labor-intensive, time-consuming, and inherently subjective, with outcomes varying significantly based on individual expertise and interpretation. The complexity of automated segmentation arises from several intrinsic limitations of ultrasound imaging. Firstly, fibroids often exhibit low contrast relative to adjacent normal tissues. Secondly, fibroids usually occupy only a small portion of the imaging area. Thirdly, the significant differences in the shape, echo characteristics and spatial position among uterine fibroids.

In literature, various techniques have been explored to solve the problem of uterine fibroid segmentation in ultrasound images. Among them, Ni et al. (2015) introduced an approach that leverages a dynamic statistical shape model to enhance the segmentation accuracy of anatomical structures. Ni et al. (2016) developed a method that incorporates the correlation among multiple target shapes as a form of prior knowledge to guide the evolution of the active contour. Zhang et al. (2023) combined the advantages of the compression and activation module and the pyramid pooling module to enhance the feature representation in the task of uterine fibroid segmentation. Liu et al. (2025a) employed 3D V-Net as the foundational architecture for their model, leveraging its strong capability in volumetric medical image segmentation. To enhance the training efficiency and guide the learning process more effectively, they incorporated a deep supervision strategy into the intermediate layers of the network. Lekshmanan Chinna and Pathrose Mary (2024) proposed an enhanced version of the bird flock optimization algorithm specifically tailored for the analysis of uterine fibroids. This improved algorithm was designed to more accurately extract relevant morphological and textural features. Cai et al. (2024) integrated MobileNetV2 with a generative adversarial network framework. This combination aimed to leverage MobileNetV2’s feature extraction capabilities while utilizing the generative power of generative adversarial network.

Furthermore, the rapid advancement of deep learning techniques in recent years has opened new avenues for improving the precision and reliability of automatic uterine fibroid segmentation. For instance, architectures such as attention mechanism (Zhou et al., 2025; Polattimur et al., 2025), multi-scale feature extractors (Agarwal et al., 2024; Hu et al., 2025), and hybrid encoder-decoder frameworks (Kumar et al., 2022; Zhu et al., 2025) have demonstrated significant potential in capturing complex anatomical structures and subtle boundary details. Among them, Ali and Xie, (2025) introduced a dynamic feature integration block designed to mitigate the semantic gap between the encoder and decoder stages in their network architecture. Zhang et al. (2025a) proposed the use of a shared global encoder to effectively capture consistent anatomical structures across varying input data. Li et al. (2025) proposed an interactive context aggregation module, which can solve the semantic inconsistency problem that often occurs when integrating multi-scale features. Huang and Xiao, (2025) introduced an advanced framework that combines efficient selective channel attention with a convolution-transformer fusion strategy. This mechanism selectively emphasizes informative channel-wise features while suppressing less relevant ones, thereby enhancing the network’s representational capacity. Liu et al. (2025b) proposed a multi-scale feature pyramid module, which incorporates an attention mechanism to enhance the model’s ability to focus on informative features across different spatial resolutions. Sun et al. (2025) introduced the multi-scale Mamba feature extraction block to enhance the network’s ability to capture rich and diverse features across multiple spatial resolutions. Deng et al. (2025) introduced a dual-branch convolutional boundary enhancement module aimed at improving the delineation of object edges in segmentation tasks. This module is composed of two parallel pathways: one dedicated to capturing semantic context, and the other focused on enhancing boundary-specific features. Xiao et al. (Xiao et al., 2025) proposed an enhanced network architecture that incorporates multi-level residual convolution within the skip connections to facilitate more effective feature propagation between the encoder and decoder. Ying et al. (2025) proposed a shape-supervised learning strategy aimed at enhancing the segmentation performance of ultrasound images by guiding the network to better capture the structural characteristics of central muscle regions. Ahmed and Lasserre, (2025) introduced a weighted multiplication fusion module tailored for breast ultrasound image analysis, aiming to enhance the quality of feature representations by mitigating the impact of inherent speckle noise.

Inspired by the challenges in ultrasound-based fibroid segmentation, we propose a novel multi-receptive attention fusion network (MAF-Net), which integrates the strengths of multi-receptive attention fusion module, dual-scale attention enhancement module, and dual-path squeeze-and-excitation enhancement module. The major contributions are summarized as follows.

1. The multi-receptive attention fusion module is embedded in the deeper layers of the encoder to improve contextual representation. By aggregating multi-scale receptive field information through attention mechanisms, this module enables the network to capture both global context and fine-grained semantic cues.
2. The dual-scale attention enhancement module is introduced within the decoder to enhance segmentation accuracy by processing image features at two distinct resolutions. Through dual-scale attention operations, this module effectively balances the integration of high-resolution structural details and low-resolution semantic context.
3. The dual-path squeeze-and-excitation enhancement module is incorporated into the skip connection to strengthen feature transmission between the encoder and decoder. Unlike conventional skip connections, DSEEM refines both channel-wise and spatial feature responses via parallel squeeze-and-excitation pathways, facilitating richer and more discriminative feature fusion across network stages.

## 2 Methods

### 2.1 Overview of MAF-net

**FIGURE 1:**
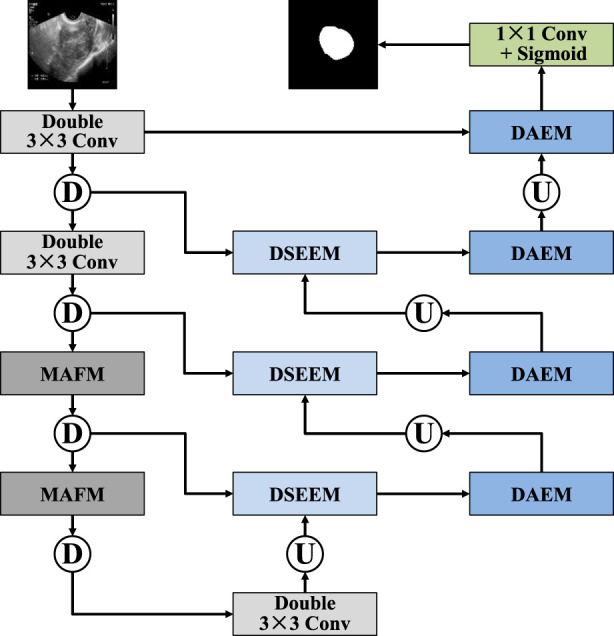
Architecture of MAF-Net.

In this section, we provide a detailed description of the overall structure of MAF-Net. This network is based on the classic U-Net architecture and consists of encoder modules, decoder modules, and improved skip connections, as shown in Figure 1. In the encoder part, MAF-Net performs feature extraction and down-sampling on the input ultrasound images layer by layer. The first two layers adopt a double-layer 3 × 3 convolution structure to extract basic features such as edge contours and textures. As the network becomes increasingly deep, the information density of the shallow layers gradually decreases, while the deep layers carry richer and more abstract semantic information. Therefore, we introduced the multi-receptive attention fusion module in the third and fourth layers of the encoder. Through the combination of different scale receptive fields and the weighted fusion of the attention mechanism, the model’s ability to perceive complex structures and blurred boundaries has been significantly enhanced. In the decoder, to fully integrate the feature information of different scales, we designed the dual-scale attention enhancement module. This module conducts parallel modeling of high-resolution and low-resolution image features at each decoding stage, which not only retains the detailed features but also incorporates the macroscopic semantic context. To address the issues of low information utilization and poor semantic consistency in the traditional U-Net skip connection structure, we introduced the dual-path squeeze-and-excitation enhancement module, which integrates the dual-path attention mechanism. It adaptively adjusts the feature responses from both the channel and spatial dimensions, and also enhances the coupling between shallow and deep features. Finally, a 1 × 1 convolutional layer followed by a sigmoid activation function is applied to the final decoder output to generate the binary segmentation map.

### 2.2 Multi-receptive attention fusion module

**FIGURE 2:**
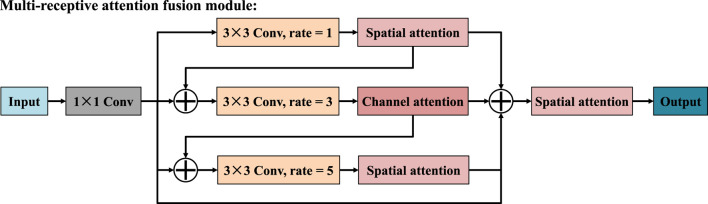
Structure of multi-receptive attention fusion module.

**FIGURE 3:**
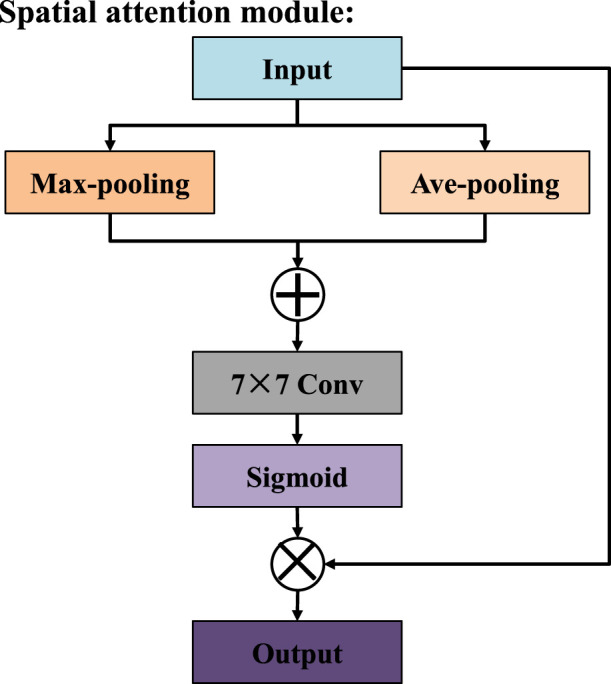
Structure of spatial attention mechanism.

**FIGURE 4:**
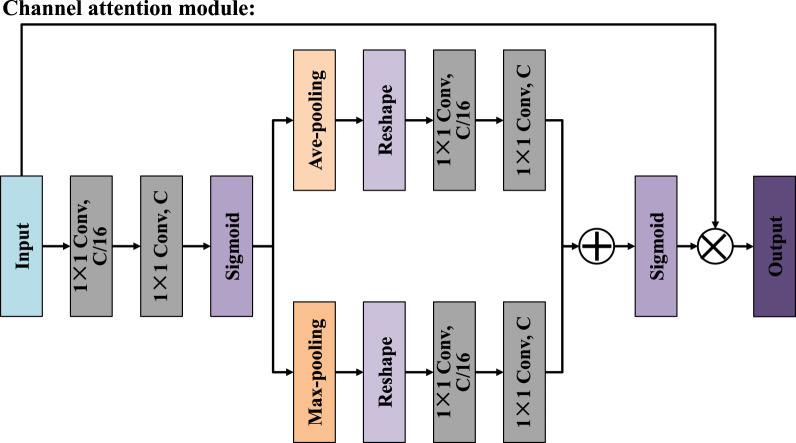
Structure of channel attention mechanism.

To address the issues of limited receptive field and difficulty in capturing global context information in traditional convolutional methods, we designed the multi-receptive attention fusion module, whose structure is shown in Figure 2. In terms of the specific structure, the input features are first subjected to a 1 × 1 convolution, which serves to reduce dimensionality and unify feature channels before further processing. Then, the resulting features are propagated through three distinct parallel branches, each of which performs a 3 × 3 convolution operation with different dilation rates, namely 1, 3, and 5. To further improve the discriminative power of the feature representation, we introduce a spatial attention mechanism (Cheng et al., 2022) in the first and third branches, as shown in Figure 3. In the second branch, the module introduces a channel attention mechanism (Cai et al., 2025), which is used to explore the dependencies between channels and generates a channel importance weight map, as shown in Figure 4. It is worth noting that MAFM not only adopts a multi-scale parallel structure but also enhances the coupling between features through an information fusion strategy across branches. Before the second branch begins, the feature map is fused with the output of the first branch. Similarly, the third branch also fuses the outputs of the first and second branches. This mechanism not only enhances the correlation between features, but also improves the optimization behavior by addressing issues such as gradient vanishing. After that, the outputs of all three branches, along with the original input features from the main path, are aggregated through a unified fusion operation. In the final stage, to further refine the aggregated feature map, an additional spatial attention module is applied. Compared with the convolutional block attention module (CBAM), the MAFM introduces a more advanced mechanism by simultaneously leveraging spatial and channel attention in a complementary fashion. By introducing multi-scale dilated convolution, MAFM effectively expands the receptive field and can capture richer semantic information at different resolutions. Moreover, the architecture incorporates a cross-branch connection strategy to reduce the risk of network degradation. This design not only maintains the integrity of features between layers but also enhances the diversity and representational ability of feature extraction.

### 2.3 Dual-scale attention enhancement module

**FIGURE 5:**
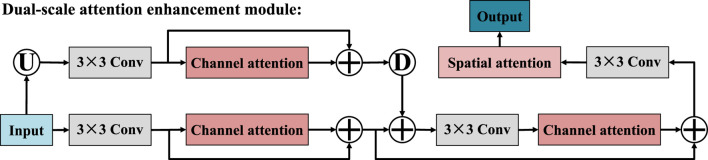
Structure of dual-scale attention enhancement module.

To enhance the feature restoration capability and semantic expression effect in the decoding stage, we designed a dual-scale attention enhancement module, as shown in Figure 5. Specifically, the DAEM starts with feature maps at two different scales: one is the original resolution feature map, and the other is its up-sampling version, which provides a broader contextual view. The feature maps of these two scales are respectively input into the parallel 3 × 3 convolution operations. After the initial convolution processing is completed, the branches of the two scales respectively introduce the channel attention mechanism to explore the dependency relationships among different channels in the feature map. Subsequently, the feature maps before and after the attention mechanism processing are fused. This fusion can retain the original structural information while incorporating the enhanced semantic features through attention. Next, the features of the two scales are further fused. This operation aims to achieve the collaborative modeling of local detail information in high-resolution images and global context information in low-resolution images. The fused feature map is then subjected to a combination of two convolutions and two attention enhancement modules. In these two convolution-attention stages, the channel attention mechanism was first adopted, and the spatial attention mechanism was introduced in the second stage. Finally, the module outputs a high-quality feature map that integrates semantic information from multiple scales, channel dimensions, and spatial dimensions, which is used to guide the subsequent segmentation prediction. Similarly, our DAEM is derived from concept of CBAM but introduces a significant advancement by processing the input image across multiple resolutions in parallel. This dual-scale strategy enables the module to simultaneously capture fine-grained local texture details and broader global contextual cues, which are both critical for accurate ultrasound image segmentation.

### 2.4 Dual-path squeeze-and-excitation enhancement module

**FIGURE 6:**
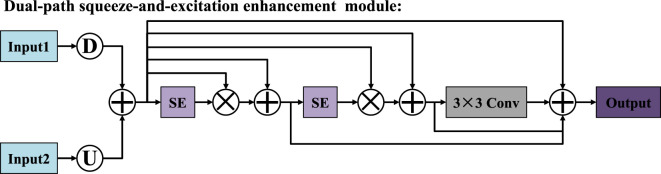
Structure of dual-path squeeze-and-excitation enhancement module.

**FIGURE 7:**
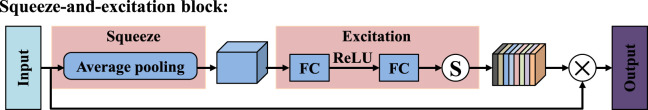
Structure of squeeze-and-excitation module.

To overcome the limitations of the traditional U-Net skip connection structure in terms of semantic consistency and feature transmission, we propose a dual-path squeeze-and-excitation enhancement module, as shown in Figure 6. This module takes the down-sampled features from the encoder (Input1) and the up-sampled features from the decoder (Input2) as its inputs. Through the fusion of multiple paths and the reinforcement of the attention mechanism, it enhances the feature expression ability. Firstly, the feature maps output by the encoder are down-sampled to unify their scales, while the feature maps output by the decoder are up-sampled to match the size of the encoder. Subsequently, these two are fused in the channel dimension to form the basic feature representation of this module. Next, the fused features are input into two cascaded squeeze-and-excitation (SE) modules (Xiong et al., 2024; Wang et al., 2025) to introduce the channel attention mechanism. The SE mechanism compresses the spatial dimensions through global average pooling to establish the dependency relationships between channels, and uses fully connected layers and activation functions to generate precise channel weights, as shown in Figure 7. After the first SE module processing, the weight map is multiplied with the base features channel by channel to complete the first attention enhancement. Subsequently, this enhancement attention feature is input into the second SE module for further deep refinement, thereby completing the channel attention modeling in the second stage. To avoid possible information attenuation and gradient transmission obstacles during the attention operation, we introduced residual connections after each attention processing. Finally, the fused feature map passes through a 3 × 3 convolution layer to further extract local features, and is deeply integrated with the first SE feature, the second SE feature, and the convolutional extracted features. Compared with the traditional SE module, which only captures channel-wise dependencies, and attention gate mechanisms that focus on spatial relevance, our proposed DSEEM integrates both types of attention across dual paths with different resolutions. Additionally, by incorporating dense connections, DSEEM allows for deeper semantic feature reuse, ensuring robust and discriminative feature representations especially suitable for challenging ultrasound scenarios.

### 2.5 Loss function

To evaluate the consistency between the predicted segmentation results and the true labels, we adopted Dice as the loss function (Fu et al., 2024; Zhang et al., 2024). The mathematical formula of Dice loss is given in Equation 1:

$$L_{dice}\left(y,p\right)=1-\frac{2\sum_{i=1}^{N}p_{i}y_{i}}{\sum_{i=1}^{N}y_{i}+\sum_{i=1}^{N}p_{i}}$$ (1)

where N is the number of pixels, $p_{i}$ and $y_{i}$ are the true labels and predicted results.

## 3 Experiments and results

### 3.1 Dataset

**FIGURE 8:**
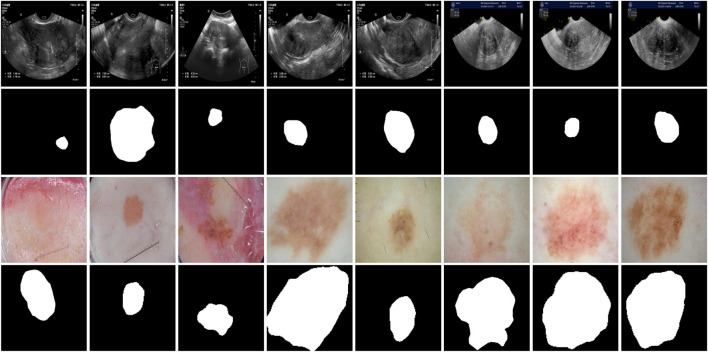
Representative examples of uterine fibroid dataset and ISIC-2018 dataset. The first and second rows are images with their corresponding annotations on the uterine fibroid dataset. The third and fourth are images with their corresponding annotations on the ISIC-2018 dataset.

To comprehensively assess the segmentation performance and generalization capability of MAF-Net, we conducted experiments on two distinct datasets: a clinical ultrasound dataset of uterine fibroid collected from Quzhou Hospital of Traditional Chinese Medicine, and the publicly available ISIC-2018 skin lesion segmentation dataset (Codella et al., 2019). The visual examples of these datasets are illustrated in Figure 8. Given the inherent complexity of the MAF-Net architecture and the constraints imposed by GPU memory, all input images were uniformly resized to 256 × 256 pixels. Furthermore, to guarantee the fairness and reproducibility of the evaluation, all experiments were conducted under identical experimental settings. The detailed summary of these datasets characteristics are provided in Table 1.

**TABLE 1:** Detailed summary of uterine fibroid dataset and ISIC-2018 dataset.

Dataset	Number	Training	Validation	Testing
Uterine fibroid dataset	1,484	891	297	296
ISIC-2018 dataset	3,694	2,594	100	1,000

Uterine fibroid dataset: The dataset was sourced from Quzhou Hospital of Traditional Chinese Medicine and comprises a total of 1,484 high-resolution ultrasound images specifically capturing uterine fibroid cases. These images were acquired under real-world clinical diagnostic settings and reflect a broad spectrum of fibroid presentations in terms of size, shape, and anatomical location. To ensure the reliability and clinical relevance of the ground truth, all images were meticulously annotated by experienced medical professionals, with manual segmentation masks delineating the fibroid regions. For the purposes of training, validation, and performance evaluation, the dataset was systematically partitioned into three subsets. Specifically, 891 images were for training, 297 images were for validation, and the remaining 296 images were designated as the independent testing.

ISIC-2018 dataset: In addition to the clinical ultrasound dataset, we also incorporated the ISIC-2018 skin lesion segmentation dataset to further validate the robustness and cross-domain generalization of our proposed method. This publicly available benchmark dataset contains a total of 3,694 dermoscopic images, each accompanied by high-quality ground truth masks that outline the lesion regions. To ensure a structured evaluation framework, the dataset was divided into three subsets: 2,594 were for training, 100 were for validation, and the remaining 1,000 were for testing.

### 3.2 Implementation details

**FIGURE 9:**
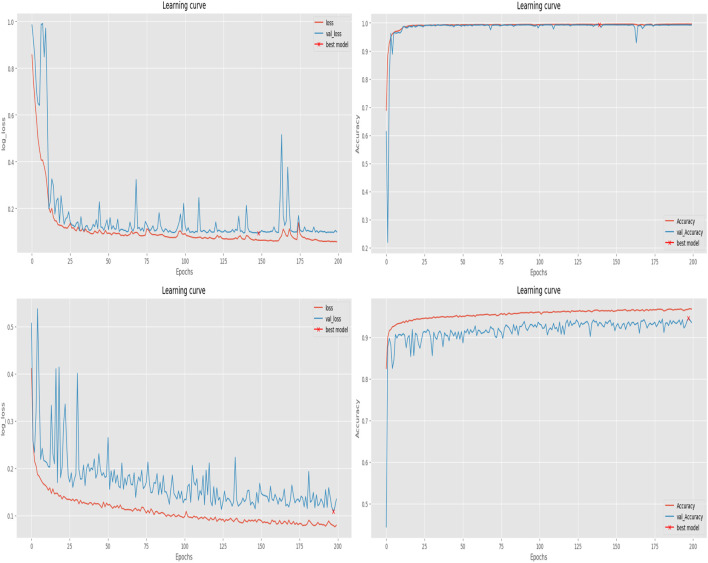
The loss and accuracy curves throughout the training and validation phases of our network. The first row is the results on the uterine fibroid dataset. The second is the results on the ISIC-2018 dataset.

**FIGURE 10:**
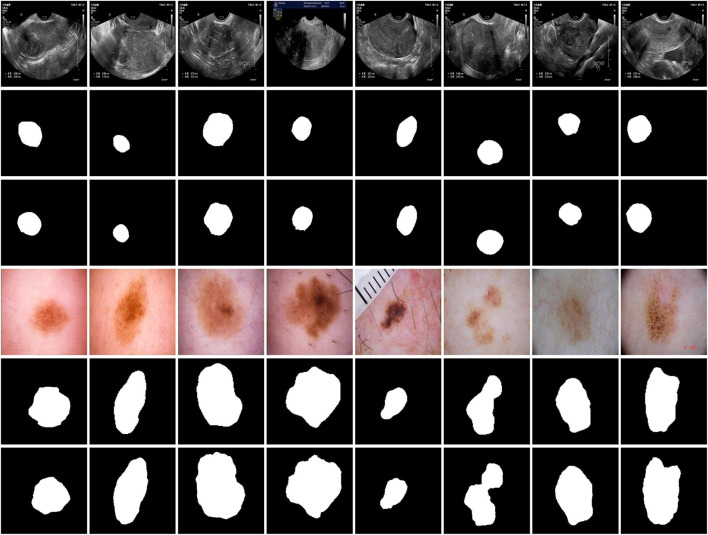
Results of MAF-Net on the uterine fibroid dataset and ISIC-2018 dataset. The first to third rows are images, their corresponding annotations and our segmentation masks on the uterine fibroid dataset. The last three rows are images, their corresponding annotations and our segmentation masks on the ISIC-2018 dataset.

The training process of MAF-Net was implemented using the TensorFlow framework on a GeForce RTX 4090 GPU with 24 GB of memory. In our experiment, we employed the Adam optimizer (Li et al., 2023) and set its initial learning rate to 0.001. Each model was trained for 200 epochs with a batch size of 16. Figure 9 illustrates the evolution of loss and accuracy metrics over the course of training and validation on two distinct datasets. The upper row presents results on the uterine fibroid dataset, where the loss curves show a rapid descent in the initial epochs followed by stable low values, indicating efficient minimization of the objective function. The close alignment between training and validation loss suggests that the model maintains good generalization without evidence of overfitting. In parallel, the accuracy curves demonstrate a sharp increase early in training, ultimately reaching a plateau above 0.95, with minimal divergence between training and validation performance. The second row displays similar trends on the ISIC-2018 dataset. Although the initial loss values differ due to dataset complexity, the overall trajectory also shows steady improvement, with smooth convergence and stable validation behavior. The accuracy curves rise consistently and maintain high levels above 0.9, again confirming the model’s robustness and adaptability across varied segmentation domains. Figure 10 showcases the qualitative segmentation results produced by MAF-Net on the uterine fibroid dataset and the ISIC-2018 dataset. These visual results highlight the model’s ability to accurately delineate lesion boundaries and preserve structural details across different medical imaging modalities.

### 3.3 Evaluation indicators

To comprehensively evaluate the segmentation performance of MAF-Net and ensure a fair comparison with several well-established algorithms, we employed five widely metrics: Dice (Selvaraj and Nithiyaraj, 2023; Li et al., 2022), Mcc (Rainio et al., 2024; Zhu, 2020), Jaccard (Yang et al., 2024; Yuan et al., 2024), Accuracy (Yang et al., 2025; Hu et al., 2025) and Recall (Zhang et al., 2025b; Xia et al., 2024). The formula for the Dice is shown in Equation 2, the formula for the Mcc is shown in Equation 3, the formula for the Jaccard is shown in Equation 4, the formula for the Accuracy is shown in Equation 5, and the formula for the Recall is shown in Equation 6:

$$Dice=\frac{2TP}{2TP+FN+FP}$$ (2)

$$Mcc=\frac{TP\times TN-FP\times FN}{\sqrt{\left(TP+FN\right)\left(TP+FP\right)\left(TN+FN\right)\left(TN+FP\right)}}$$ (3)

$$Jaccard=\frac{TP}{TP+FN+FP}$$ (4)

$$Accuracy=\frac{TP+TN}{TP+TN+FN+FP}$$ (5)

$$Recall=\frac{TP}{TP+FN}$$ (6)

### 3.4 Ablation experiments

To further validate the effectiveness of each proposed module within the MFA-Net architecture, we conducted comprehensive ablation experiments on the uterine fibroid dataset. As summarized in Table 2, the Baseline model, which excludes all proposed enhancement modules, achieves Dice of 0.8993, Mcc of 0.8957, Jaccard of 0.8178, Accuracy of 0.9912, and Recall of 0.8712. When the multi-receptive attention fusion module is integrated into the Baseline, all five metrics show noticeable improvement, with the Dice increasing to 0.9076, the Mcc increasing to 0.9038, the Jaccard reaching to 0.8311, the Accuracy reaching to 0.9919, while the Recall reaching to 0.9014. This indicates that MAFM effectively enhances the network’s ability to capture multi-scale contextual information and refine feature attention. Similarly, incorporating the dual-scale attention enhancement module yields a moderate performance gain, pushing the Dice to 0.9044, the Mcc to 0.9003, the Jaccard to 0.8259, the Accuracy to 0.9916, and the Recall to 0.8956. This improvement suggests that DAEM contributes to more precise localization and boundary refinement by adaptively focusing on different spatial scales. Adding the dual-path squeeze-and-excitation enhancement module also leads to a clear performance boost over the baseline, with Dice of 0.9071, Mcc of 0.9031, Jaccard of 0.8301, Accuracy of 0.9918 and Recall of 0.8956. Most notably, when all three modules (MAFM + DAEM + DSEEM) are combined, the model achieves the highest performance across all metrics: Dice of 0.9126, Mcc of 0.9089, Jaccard of 0.8394, Accuracy of 0.9924, and Recall of 0.9016. These results indicate that each module can bring about unique benefits, and their coordinated integration can achieve the best segmentation accuracy. Meanwhile, Table 2 also presents the parameters and running time when integrating each module into the basic network. Specifically, the baseline model starts with 2.06 million parameters and a per-step inference time of 45 ms, representing a lightweight architecture. After adding the MAFM module, the parameter count increases modestly to 2.41M, and the time per step rises slightly to 49 ms, suggesting that MAFM enhances feature representation with minimal computational overhead. In contrast, introducing DAEM leads to a more noticeable increase in inference time, from 45 ms to 103 ms, despite the parameter count only increasing to 2.62M. This indicates that although the DAEM module has a relatively compact structure, the operations it performs internally involve a high level of computational intensity. The inclusion of DSEEM increases the parameter count to 3.01M and inference time to 52 ms. Finally, the complete model incorporating MAFM + DAEM + DSEEM has the largest parameter size of 3.56M and a per-step time of 54 ms. Notably, even with all modules combined, the time increase relative to the baseline is only 9 ms, showing that the overall architecture maintains high computational efficiency while enabling stronger feature learning.

**TABLE 2:** Ablation experiments on the uterine fibroid dataset.

Method	Dice	Mcc	Jaccard	Accuracy	Recall	Parameter (M)	Time (ms/step)
Baseline	0.8993	0.8957	0.8178	0.9912	0.8712	2.06	45
Baseline + MAFM	0.9076	0.9038	0.8311	0.9919	0.9014	2.41	49
Baseline + DAEM	0.9044	0.9003	0.8259	0.9916	0.8956	2.62	103
Baseline + DSEEM	0.9071	0.9031	0.8301	0.9918	0.9013	3.01	52
Baseline + MAFM + DAEM + DSEEM	0.9126	0.9089	0.8394	0.9924	0.9016	3.56	54

### 3.5 Comparative experiments

#### 3.5.1 Experiments on the uterine fibroid dataset

To comprehensively evaluate the capability of MAF-Net on the uterine fibroid dataset, we carried out a series of experiments involving several methods. The benchmarked approaches include SE-U-Net (Jiang et al., 2021), CLNet (Zheng et al., 2021), RMAU-Net (Jiang et al., 2023), SegNet (Badrinarayanan et al., 2017), SSA-UNet (Jiang et al., 2024), and AttUNet (Oktay et al., 2018). The quantitative assessment of the five key indicators are summarized in Table 3. Among the evaluated models, SE-U-Net and CLNet record the lowest performance, with Dice of 81.70% and 83.50%, Mcc of 81.13% and 82.87%, Jaccard of 69.14% and 71.72%, Accuracy of 0.9912 and 0.9906, Recall of 0.8808 and 0.8644. These results suggest a limited capacity in capturing complex lesion boundaries and spatial details, likely due to inadequate contextual modeling. Slightly outperforming CLNet, SSA-UNet and SegNet report moderate improvements but still fall short of competitive accuracy Notably, RMAU-Net and AttUNet exhibit more competitive performance, with Dice coefficients of 86.86% and 87.86%, respectively. The integration of residual learning and attention mechanisms in these models contributes to more refined feature representations and improved lesion delineation. Despite these strong performances, the proposed MAF-Net surpasses all competing approaches, attaining the highest segmentation accuracy across all evaluation criteria: Dice of 91.26%, Mcc of 90.89%, Jaccard of 83.94%, Accuracy of 0.9924 and Recall of 0.9016. This superior performance can be attributed to MAF-Net’s well-crafted architectural design, which synergistically combines the multi-receptive attention fusion module, dual-scale attention enhancement module, and dual-path squeeze-and-excitation enhancement module.

**TABLE 3:** Comparative experiments on the uterine fibroid dataset.

Method	Dice	Mcc	Jaccard	Accuracy	Recall
SE-U-Net (Jiang et al., 2021)	0.8170	0.8113	0.6914	0.9912	0.8808
CLNet (Zheng et al., 2021)	0.8350	0.8287	0.7172	0.9906	0.8644
RMAU-Net (Jiang et al., 2023)	0.8686	0.8633	0.7685	0.9905	0.8746
SegNet (Badrinarayanan et al., 2017)	0.8558	0.8509	0.7498	0.9888	0.8336
SSA-UNet (Jiang et al., 2024)	0.8486	0.8429	0.7379	0.9909	0.8695
AttUNet (Oktay et al., 2018)	0.8786	0.8736	0.7840	0.9917	0.8720
MAF-Net	0.9126	0.9089	0.8394	0.9924	0.9016

**FIGURE 11:**
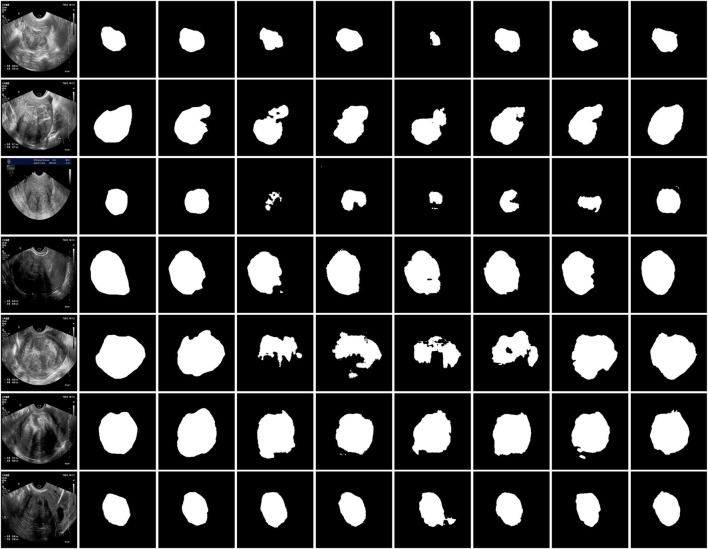
Qualitative comparison on the uterine fibroid dataset. The first to second rows are images and their corresponding annotations. The third to last are results of SE-U-Net, CLNet, RMAU-Net, SegNet, SSA-UNet, AttUNet and MAF-Net.

Figure 11 illustrates the qualitative comparison results on the uterine fibroid dataset across several state-of-the-art models. The first and second columns display the original ultrasound images and their corresponding ground truth annotations. The subsequent columns present the predicted segmentation masks generated by SE-U-Net, CLNet, RMAU-Net, SegNet, SSA-UNet, AttUNet, and the proposed MAF-Net. Among all models, SE-U-Net and CLNet demonstrate the least satisfactory performance. Its predictions are often incomplete, with fragmented and under-segmented regions that fail to align with the actual fibroid boundaries. SSA-UNet and SegNet show modest improvements, yet their segmentations still suffer from noise, discontinuities, and misaligned contours. RMAU-Net and AttUNet produce more coherent results, with better overall shape conformity and partial boundary accuracy. However, in several instances, their masks remain either overly smooth or slightly under-extended. In comparison, the proposed MAF-Net consistently achieves the most accurate and complete segmentation across all test samples. It excels at delineating fibroid regions with clear, smooth contours that closely match the ground truth. Overall, the qualitative results in Figure 11 clearly highlight MAF-Net’s advantage in capturing fine-grained structural details while maintaining high segmentation fidelity.

#### 3.5.2 Experiments on the ISIC-2018 dataset

**FIGURE 12:**
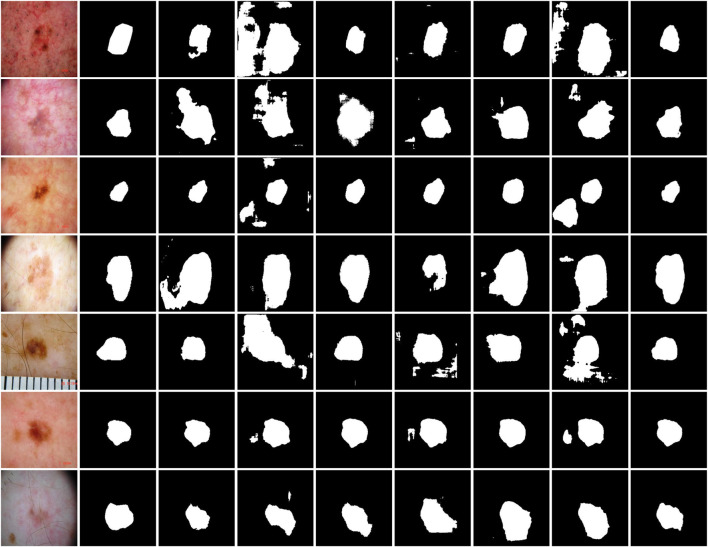
Qualitative comparison on the ISIC-2018 dataset. The first to second rows are images and their corresponding annotations. The third to last are results of SE-U-Net, CLNet, RMAU-Net, SegNet, SSA-UNet, AttUNet and MAF-Net.

Table 4 provides a detailed quantitative comparison of MAF-Net with several contemporary segmentation networks on the ISIC-2018 dataset. Among all methods evaluated, MAF-Net achieves the highest performance across all metrics, attaining Dice of 86.24%, Mcc of 81.56%, Jaccard of 76.52%, Accuracy of 0.9251 and recall of 0.8304. In contrast, SegNet yields the lowest performance, with Dice, Mcc, Jaccard, Accuracy and Recall of 81.73%, 75.02%, 69.50%, 91.07% and 82.97, respectively. This performance gap indicates significant limitations in its ability to capture intricate lesion details. Other models, such as SE-U-Net, CLNet, and RMAU-Net, deliver marginally better results, with Dice values close to 83.5% and Jaccard scores ranging between 72.17% and 72.27%. SSA-UNet achieves slightly improved results, reaching Dice of 84.00%, Mcc of 78.08%, Jaccard of 73.03%, Accuracy of 91.51% and Recall of 82.93%. However, the segmentation accuracy remains inferior to MAF-Net, particularly in terms of overlap with ground truth. Similarly, AttUNet performs well in utilizing the attention mechanism, but its Dice score is only 82.88% and fails to achieve competitive Jaccard, Mcc, Accuracy and Recall. To visually corroborate the numerical evaluation, Figure 12 presents a qualitative comparison of segmentation results across the same models. Visual inspection reveals that SegNet frequently produces coarse or overly smoothed masks. Predictions from SE-U-Net, CLNet, and RMAU-Net are more refined but still exhibit noise and partial under-segmentation in several cases. The SSA-UNet and AttUNet can generate clearer segmentation maps, but they still have shortcomings in terms of boundary clarity and sensitivity to small or irregular structures. By comparison, MAF-Net consistently produces precise and complete masks that closely match the annotated regions. In summary, both the quantitative results in Table 4 and the visual comparisons in Figure 12 confirm the effectiveness of MAF-Net in skin lesion segmentation. It not only outperforms existing architectures in metric-based evaluation but also exhibits superior visual quality and lesion localization in complex real-world cases.

**TABLE 4:** Comparative experiments on the ISIC-2018 dataset.

Method	Dice	Mcc	Jaccard	Accuracy	Recall
SE-U-Net (Jiang et al., 2021)	0.8333	0.7749	0.7227	0.9201	0.8287
CLNet (Zheng et al., 2021)	0.8354	0.7765	0.7226	0.9139	0.8239
RMAU-Net (Jiang et al., 2023)	0.8352	0.7764	0.7217	0.9200	0.8213
SegNet (Badrinarayanan et al., 2017)	0.8173	0.7502	0.6950	0.9107	0.8279
SSA-UNet (Jiang et al., 2024)	0.8400	0.7808	0.7303	0.9151	0.8293
AttUNet (Oktay et al., 2018)	0.8288	0.7653	0.7121	0.9125	0.8199
MAF-Net	0.8624	0.8156	0.7652	0.9251	0.8304

#### 3.5.3 Experiments of dilation rate in the MAFM

To investigate the impact of different dilation rate combinations within the multi-receptive attention fusion module, we conducted a comprehensive set of experiments on the uterine fibroid dataset, as summarized in Table 5. Among all tested configurations, the dilation rate combination of (1, 3, 5) achieved the highest overall performance, with Dice of 0.9126, Mcc of 0.9089, Jaccard of 0.8394 and Accuracy of 0.9924, outperforming other settings across multiple evaluation metrics. Specifically, the dilation rate of 1 enables the model to capture fine-grained spatial details, while dilation rates of 3 and 5 effectively expand the receptive field to aggregate multi-scale contextual information without causing gridding artifacts or excessive sparsity in the feature map. Compared to larger dilation combinations (e.g (1, 3, 8) or (1, 4, 8)) (1, 3, 5) avoids over-dilated convolutions that may result in missing critical structure boundaries, as reflected in their lower performance scores. Therefore, the selection of (1, 3, 5) is empirically justified and demonstrates a well-rounded ability to integrate multi-scale features.

**TABLE 5:** Experiments of dilation rate in the MAFM on the uterine fibroid dataset.

Dilation rate	Dice	Mcc	Jaccard	Accuracy	Recall
(1, 2, 3)	0.9106	0.9067	0.8361	0.9922	0.9066
(1, 2, 4)	0.9100	0.9062	0.8350	0.9922	0.9065
(1, 2, 5)	0.9086	0.9047	0.8327	0.9920	0.9080
(1, 2, 6)	0.9124	0.9087	0.8392	0.9924	0.9085
(1, 2, 7)	0.9088	0.9049	0.8331	0.9920	0.9122
(1, 2, 8)	0.9093	0.9055	0.8340	0.9921	0.8960
(1, 3, 4)	0.9080	0.9042	0.8318	0.9918	0.9253
(1, 3, 5)	0.9126	0.9089	0.8394	0.9924	0.9016
(1, 3, 6)	0.9082	0.9044	0.8321	0.9919	0.8995
(1, 3, 7)	0.9075	0.9038	0.8311	0.9920	0.8905
(1, 3, 8)	0.9020	0.8985	0.8228	0.9915	0.8779
(1, 4, 5)	0.9111	0.9074	0.8370	0.9923	0.9039
(1, 4, 6)	0.9087	0.9048	0.8329	0.9919	0.9108
(1, 4, 7)	0.9073	0.9037	0.8307	0.9920	0.8833
(1, 4, 8)	0.9061	0.9023	0.8285	0.9919	0.8921
(1, 5, 6)	0.9064	0.9028	0.8295	0.9919	0.8915
(1, 5, 7)	0.9116	0.9080	0.8378	0.9924	0.8966
(1, 5, 8)	0.9099	0.9061	0.8349	0.9921	0.9108
(1, 6, 7)	0.9085	0.9047	0.8326	0.9920	0.9036
(1, 6, 8)	0.9070	0.9031	0.8302	0.9918	0.8961
(1, 7, 8)	0.9033	0.8994	0.8242	0.9915	0.8965

#### 3.5.4 Experiments of path selection in the DSEEM

To validate the effectiveness of the dual-path design in the dual-path squeeze-and-excitation enhancement module, we conducted comparative experiments under three different configurations: (1) dual-path, (2) single-path using Input1 only, and (3) single-path using Input2 only. The quantitative results on the uterine fibroid dataset are summarized in Table 6. Among the three configurations, the dual-path variant achieved the best overall performance, with Dice of 0.9126, Mcc of 0.9089, Jaccard of 0.8394, Accuracy of 0.9924, and Recall of 0.9016. These results clearly demonstrate that simultaneously incorporating both feature streams (Input1 and Input2) enables more comprehensive and complementary feature representation. In contrast, the single-path (Input1) configuration resulted in significantly lower performance across all metrics, with Dice dropping to 0.7474 and Jaccard to 0.5979, suggesting that this path alone lacks sufficient contextual or semantic information to accurately localize lesion regions. The single-path (Input2) setting performed moderately better, with Dice of 0.8888 and Jaccard of 0.8005, but still failed to match the performance of the dual-path design. This result indicates that while Input2 carries more informative or higher-level features than Input1, it still benefits significantly from the complementary support of the other pathway. Overall, these findings confirm that the dual-path architecture in DSEEM is not merely additive, but effectively leverages multi-level feature fusion to enhance the network’s capacity in learning discriminative and semantically rich representations.

**TABLE 6:** Experiments of path selection in the DSEEM on the uterine fibroid dataset.

Path selection	Dice	Mcc	Jaccard	Accuracy	Recall
dual-path	0.9126	0.9089	0.8394	0.9924	0.9016
Single-path (Input1)	0.7474	0.7380	0.5979	0.9793	0.7186
Single-path (Input2)	0.8888	0.8844	0.8005	0.9904	0.8726

#### 3.5.5 Experiments of optimizer selection

To explore the impact of different optimization strategies on MAF-Net, we conducted a comparative experiment using five commonly used optimizers on the uterine fibroid dataset, as shown in Table 7. Among all the tested optimizers, Adam performed the best overall, with Dice of 91.26%, Mcc of 90.89%, Jaccard of 83.94%, Accuracy of 99.24, and recall of 90.16%. These results indicate that Adam’s ability to dynamically adjust the learning rate for each parameter during the training process is particularly outstanding, which helps accelerate the convergence speed and make the optimization process more stable. The performance of RMSprop is also excellent, with Dice of 90.74%, Mcc of 90.35%, Jaccard of 83.09%, Accuracy of 99.19%, and Recall of 89.44%. Although it is close to Adam in terms of segmentation accuracy, its slightly inferior performance may be attributed to the slightly poorer balance effect in terms of convergence speed and stability when dealing with different feature scales. Adamax is a variant of Adam based on the infinity norm, with Dice of 89.39%, Mcc of 88.98%, Jaccard of 80.92%, Accuracy of 99.08% and 88.00%. Although it retains many of the advantages of Adam, its sensitivity to rare gradients is relatively low, which may lead to poor parameter update effects in tasks requiring fine spatial details. Adagrad is an optimizer that dynamically adjusts the learning rate based on the frequency of parameter updates. However, its performance is significantly inferior, with Dice of 79.34%, Mcc of 79.68%, Jaccard of 66.42%, 98.37% and 67.90%. SGD is the most basic optimizer tested, and its results are clearly the worst. The Dice is 31.05%, the Mcc is 29.10%, while the Jaccard is only 18.48%. Therefore, for the segmentation framework of the uterine fibroid dataset, Adam is undoubtedly the most effective optimizer.

**TABLE 7:** Experiments of optimizer selection on the uterine fibroid dataset.

Optimizer	Dice	Mcc	Jaccard	Accuracy	Recall
Adam	0.9126	0.9089	0.8394	0.9924	0.9016
Adagrad	0.7934	0.7968	0.6642	0.9837	0.6790
Adamax	0.8939	0.8898	0.8092	0.9908	0.8800
RMSprop	0.9074	0.9035	0.8309	0.9919	0.8944
SGD	0.3105	0.2910	0.1848	0.9124	0.4733

#### 3.5.6 Experiments of computational efficiency

As presented in Table 8, we evaluate the computational efficiency of MAF-Net in comparison with these models. Among all models, SE-U-Net exhibits the smallest parameter count (1.87 M) and fastest inference speed (42 ms/step), reflecting its lightweight design. However, its simplicity may come at the cost of limited feature representation capacity. Although CLNet is highly functional, it has the largest number of parameters (7.73 M), and due to its optimized architecture, its inference time remains relatively efficient (50 ms/step). In contrast, AttUNet has an even larger model size (8.49 M) and a slower inference time (61 ms/step), highlighting the computational burden introduced by attention mechanisms when not efficiently designed. RMAU-Net and SSA-UNet present moderate parameter sizes (2.27 M and 2.33 M), yet RMAU-Net’s inference time reaches 62 ms/step, likely due to recursive or multi-scale operations, while SSA-UNet maintains a more balanced runtime of 48 ms/step. Compared to these models, our MAF-Net strikes a favorable balance between complexity and speed. With 3.56M parameters, it achieves an inference time of 54 ms/step, which is significantly lower than that of AttUNet and RMAU-Net. In summary, MAF-Net demonstrates competitive computational efficiency, offering a good trade-off between model size, runtime performance, and segmentation accuracy.

**TABLE 8:** Experiments of computational efficiency on the uterine fibroid dataset.

Method	Parameter (M)	Time (ms/step)
SE-U-Net (Jiang et al., 2021)	1.87	42
CLNet (Zheng et al., 2021)	7.73	50
RMAU-Net (Jiang et al., 2023)	2.27	62
SegNet (Badrinarayanan et al., 2017)	2.80	48
SSA-UNet (Jiang et al., 2024)	2.33	48
AttUNet (Oktay et al., 2018)	8.49	61
MAF-Net	3.56	54

### 3.6 Limitations

**FIGURE 13:**
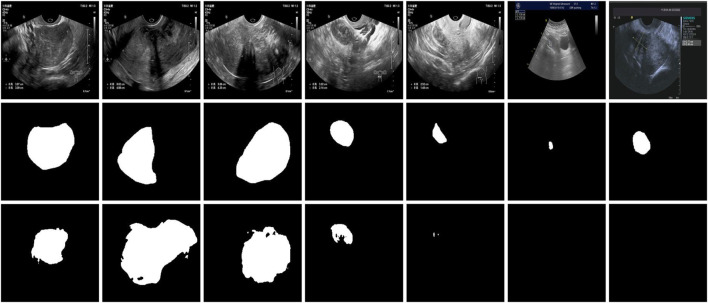
Failure cases from the uterine fibroid dataset. The first and second rows are images with their corresponding annotations on the uterine fibroid dataset. The third row is the results of MAF-Net.

Figure 13 presents several failure cases from the uterine fibroid dataset of the proposed MAF-Net. In some cases (e.g., columns 2 and 3), the predicted masks are noticeably larger than the ground truth annotations, indicating over-segmentation. This may be attributed to blurred lesion boundaries or low-contrast regions in the ultrasound images, which confuse the model and lead it to mistakenly include surrounding normal tissues. Conversely, under-segmentation is evident in columns 1 and 4, where the model identifies only a small portion of the lesion or fails to detect it almost entirely. This typically occurs when the lesion is small, indistinct from the background, or has poor contrast, making it difficult for the network to capture complete contextual information. Mis-segmentation is observed in cases such as columns 5, 6, and 7, where the predicted regions are significantly misaligned with the actual lesion locations. These failures may result from the presence of structures with similar textures or intensities, which mislead the model into segmenting anatomically irrelevant regions. Overall, these cases highlight the challenges posed by low-contrast lesions, boundary ambiguity, and anatomical variability in ultrasound images. In the future, we will introduce boundary perception mechanisms, enhance multi-scale feature extraction, or utilize auxiliary supervision to further improve the robustness and accuracy of MAF-Net in complex scenarios.

## 4 Conclusion

In this study, we proposed the MAF-Net deep learning framework, which was specifically designed for precise segmentation of uterine fibroids in ultrasound imaging. Specifically, by utilizing a unified encoder-decoder architecture, MAF-Net combined the multi-receptive attention fusion module, the dual-path squeeze-and-excitation enhancement module, and the dual-scale attention enhancement module, enabling it to effectively handle the inherent noise, boundary blurring, and scale variations in clinical ultrasound data. Extensive validation on the real-world uterine fibroid dataset demonstrated that MAF-Net consistently outperforms existing models in key performance metrics. The evaluation on the ISIC-2018 dataset further confirmed its strong generalization ability. Additionally, ablation studies emphasized the synergy of each architectural module, which collectively enhanced the accuracy and robustness. Overall, MAF-Net provided a reliable, accurate, and clinically applicable automatic segmentation solution for the ultrasound diagnostic workflow.

## Data Availability

The raw data supporting the conclusions of this article will be made available by the authors, without undue reservation.
